# A Bioassay-Based Approach for the Batch-To-Batch Consistency Evaluation of Xuesaitong Injection on a Zebrafish Thrombosis Model

**DOI:** 10.3389/fphar.2021.623533

**Published:** 2021-03-08

**Authors:** Xiangwei Ma, Yanyu Chen, Shumin Jiang, Xiaoping Zhao

**Affiliations:** ^1^ College of Pharmaceutical Sciences, Zhejiang Chinese Medical University, Hangzhou, China; ^2^ School of Basic Medical Sciences, Zhejiang Chinese Medical University, Hangzhou, China; ^3^ Academy of Chinese Medical Science, Zhejiang Chinese Medical University, Hangzhou, China

**Keywords:** Chinese medicine, zebrafish thrombosis model, batch-to-batch consistency, Xuesaitong injection, quality control

## Abstract

Quality control of Chinese medicine (CM) is mainly based on chemical testing, which sometimes shows weak correlation to pharmacological effects. Thus, there is a great demand to establish bioactivity-based assays to ensure the quality of CM. The aim of the present study was to establish a bioassay-based approach to evaluate the biological activity of Xuesaitong injection (XST) based on an *in vivo* zebrafish model. Zebrafish larvae with arachidonic acid (AA)-induced thrombus were applied to evaluate anti-thrombosis effects of XST and explore the potential mechanism of XST. Analysis of major components in normal and abnormal XST samples was performed by high performance liquid chromatography (HPLC). The results indicate that XST could significantly restore heart red blood cells (RBCs) intensity of thrombotic zebrafish in a dose-dependent manner, whilst decreasing RBCs accumulation in the caudal vein. The results were confirmed using a green fluorescence protein (GFP)-labeled zebrafish thrombosis model. Moreover, we could show that XST downregulates the expression of the *fibrinogen alpha chain* (*fga*) gene to inhibit the coagulation cascade during the process of thrombosis in zebrafish. Notoginsenoside R_1,_ ginsenoside Rg_1_, ginsenoside Rb_1_ and ginsenoside Rd, which were considered to be the major components of XST, also showed moderate anti-thrombosis efficacy. Further results showed that the zebrafish thrombosis model could efficiently distinguish five abnormal batches of XST from 24 normal batches. Furthermore, the inhibition rates of different batches were correlated with the content level of major components. Our results suggested that the proposed zebrafish thrombosis model could be successfully used to evaluate the batch-to-batch consistency of XST, which provided an alternative way for the quality control of CM.

## Introduction

Traditional Chinese medicine (TCM) is gaining increasing attention worldwide for its distinguished efficacy and minimum side effects (Shi et al., 2018). As a TCM preparation, CM is a combination of TCM theory and modern technology, which has the advantages of high stability, convenient for transport and storage. Thus, the quality assessment of CM is an urgent task in the process of the standardization and internationalization of TCM (Wang et al., 2016; Wang et al., 2018).

Diverse analysis methods have been used for quality evaluation of CM, by determining the content of several active components, such as GC, HPLC, liquid chromatography-mass spectrometry (LC-MS), near-infrared spectroscopy (NIRS), and Raman spectroscopy (RS). A promising method for the quality control of *Astragalus mongholicus* Bunge (Astragali radix) and its products was developed by the SIM mode of ultra-performance liquid chromatography (UPLC-QDA) (Zhao et al., 2018). Wang developed a method based on HPLC coupled with a photodiode array detector for the quality evaluation of Yin Chen Hao Tang extract, which was successfully applied in the analysis of 12 different batch samples (Wang et al., 2008b). UPLC coupled with quadrupole time-of-flight mass spectrometry (UHPLC-QTOF-MS) was employed to the quality assessment of Yin-Qiao-Jie-Du tablets (He et al., 2016). A quantitative analysis model was employed to the rapid quality control of Tongkang tablets by NIRS (Pan et al., 2017).

The batch-to-batch consistency is an important part of quality evaluation of CM. Mid-infrared and ultra violet spectroscopic fingerprints were integrated for quality consistency of Weibizhi tablets (Liu et al., 2016). Li combined in-line near-infrared spectroscopy with a multivariate statistical process control method, simultaneously developed a multi-way principal component analysis model and adopted multivariate control charts for the real-time monitoring of TCM products (Li et al., 2016).

Nonetheless, the content level determination of single or multiple components cannot completely evaluate the effectiveness of CM (Wu et al., 2018), thus leaving uncertainty for quality assessment as well as therapeutic consistency. Biological assays were suggested to ensure the therapeutic consistency of botanical drug products in Botanical Drug Development Guidance for Industry promulgated by the Food and Drug Administration (FDA) (FDA, 2016). Zhang (Zhang et al., 2014) combined chemical fingerprints with biological fingerprints to detect the quality fluctuation of SHL, a herbal injection. The antioxidant capability was tested for the stability evaluation of Danhong injection based on human umbilical vein endothelial cells (HUVECs) (Li et al., 2017). Lam proposed that the quality control of a herbal product should be based on its pharmacological mechanism. They developed a platform which consisted of 18 luciferase reporter cell lines and two enzymatic assays to assess Chinese formulations and commercial products (Lam et al., 2018). Consequently, we propose the development of a model organism-based test for the consistency assessment of CM injection.

The zebrafish (*Danio rerio*), an aquatic vertebrate, is currently becoming a prevalent model organism, which is widely applied for biomedical research, toxicology studies, as well as drug discovery (Peterson and MacRae, 2012; Kalueff et al., 2014; MacRae and Peterson, 2015). Zebrafish have multiple advantages including high homology to mammals, higher fecundity, rapid development of embryos, and transparent embryos and larvae (Loehr and Hammerschmidt, 2011; Stewart et al., 2014). Zebrafish have been employed for TCM research in many aspects, for example, active ingredients screening, and toxicity evaluation (Tu et al., 2016; Yang et al., 2017). The zebrafish is a useful model in thrombosis research (Gibbins and Mahaut-Smith, 2012), because platelet function shares many characteristics with humans, such as GPIIb-IIIa, coagulation factors, and arachidonic acid metabolism enzymes (Jagadeeswaran et al., 1999; Lang et al., 2010). Thus, it is meaningful to apply the zebrafish thrombosis model for the quality evaluation of CM. Our research group has successfully established a biological quality assessment assay of Danhong injection by a thrombosis model of zebrafish (Qi et al., 2017).

*Panax notoginseng* (Burk.) F. H. Chen is documented from Compendium of Materia Medica to Chinese Pharmacopoeia, which has the functions of removing blood stasis and hemostasis, reducing swelling, and relieving pain. The main component of *Panax notoginseng* (Burk.) F. H. Chen is Panax notoginseng saponin (PNS). Xuesaitong injection (XST) is a freeze-dried powder injection, which is made from the PNS, is extensively applied for the treatment of cardiovascular and cerebrovascular diseases in modern medicine (Wen and Liu, 2007; Chen et al., 2014).

Our group has made a series of contributions to the chemical and pharmacological study of XST. Notoginsenoside R_1_, ginsenoside Rg_1_, ginsenoside Rb_1_, ginsenoside Rd, and ginsenoside Re are approximately 85% of the total components in XST, whose combination possesses a considerably similar efficacy to XST in the myocardial infarction rat model (Wang et al., 2014). Wang constructed a compound-target-pathway network to explore the mechanism of XST against myocardial infarction (Wang et al., 2013). Zhao demonstrated that the cardioprotective effect of XST against ischemia/reperfusion (I/R) injury was related to myocardial energy metabolism as well as oxidative stress (Zhao et al., 2017). Wang elucidated that anti-platelet aggregation and anti-inflammation were the main mechanisms of XST involved in preventing ischemia-reperfusion injury (Wang et al., 2015). Ma established a biological evaluation method for the quality assessment of XST based on anti-inflammatory activity (Ma et al., 2019). In addition, we developed a chemical analysis method for the quality evaluation of XST (Yao et al., 2011), however there was a lack of biological assays, which was directly correlated to its pharmacological effects to ensure therapeutic consistency. In this work, a reliable method based on a zebrafish thrombosis model was developed for the quality and batch-to-batch consistency testing of XST.

## Materials and Methods

### Materials

Dimethyl sulfoxide (DMSO), tricaine, 1-phenyl-2-thio-urea (PTU), and *o*-dianisdine were purchased from Sigma-Aldrich Inc. (St. Louis, MO, United States). AA was provided by the Shanghai Yuanye Bio-Technology Co., Ltd. (Shanghai, China). Ginsenoside Rg_1_ (150823, purity ≥ 98%), ginsenoside Rb_1_ (120120, purity ≥ 98%), ginsenoside Re (120723, purity ≥ 98%), ginsenoside Rd (120507, purity ≥ 98%), and notoginsenoside R_1_ (120821, purity ≥ 99%) were purchased from the Ronghe Pharmaceutical Technology Development Co., Ltd. (Shanghai, China). Aspirin was bought from the Dalian Meilun Biological Technology Co., Ltd. (Dalian, China). Glycerol and 4% paraformaldehyde fix solution were obtained from the Sangon Biotech Co., Ltd. (Shanghai, China). Ultrahigh-purity water was produced by ELGA purelab (High Wycombe, United Kingdom). Acetonitrile and methanol were obtained from Merck KGaA (HPLC grade, Darmstadt, Germany). Acetic acid was obtained from ROE Scientific Inc. (HPLC grade, Newark, United States). The 24 normal batches of XST (lyophilized powder) were provided by the Heilongjiang Zhenbaodao Pharmaceutical Co., Ltd. (Heilongjiang, China), and the batch numbers were F704001a1, F704029a1, F704019a3, F704003a3, F701003a2, F704024a4, F704016a4, F704015a3, F701009a1, F704002a2, F704030a2, F704038a2, F704031a3, F704017a1, F701011a3, F704004a4, F704020a4, F704021a1, F704033a1, F704023a3, F704026a2, F701007a3, F704022a2, and F704025a1.

### Zebrafish care, Maintenance, and Breeding

Adult wild-type Tuebingen (TU) strain zebrafish and transgenic LCR-GFP zebrafish were bought from the China Zebrafish Resource Center (Wuhan, China). Zebrafish were cultured in a light (14:10 light/dark cycle) and temperature controlled aquaculture facility. Zebrafish were fed with alive brine shrimp twice a day and dry flake once per day. Embryos and adult fish were maintained in fish water (deionized water containing 0.3% Instant Ocean Salt, temperature at 28.5°C, pH 6.9–7.2, hardness approximately 90 mg/L CaCO_3_, conductivity 500–550 µs/cm).

The male and female adult fish were placed in a mating tank at a ratio of 1:1 (or 1:2) and separated by a baffle. The baffle was removed to allow free mating the next day. Two hours later, the embryos were collected and washed with fish water to remove dysplastic embryos and debris. The embryos were incubated in fish water containing methylene blue (inhibiting the growth of bacteria) and transferred to a 28.5°C incubator for cultivation. The fish water was changed and 2.5 μmol/L of PTU was added to inhibit melanin growth at 1dpf and 2dpf (days past fertilization, dpf).

### Reagent Preparation

AA, XST, aspirin, notoginsenoside R_1_, ginsenoside Rg_1_, ginsenoside Re, ginsenoside Rb_1,_ and ginsenoside Rd were dissolved in DMSO as stock solution, and diluted in fish water containing PTU. The final concentration of XST was 400 µg/ml; the final concentration of ginsenoside Rg_1_, ginsenoside Rb_1_, ginsenoside Re, and notoginsenoside R_1_ was 200 µmol/L, the final concentration of ginsenoside Rd was 100 µmol/L.

The abnormal batches were prepared by excluding or reducing the content of one or two components in XST (notoginsenoside R_1_ accounts for 10.85%, ginsenoside Rg_1_ accounts for 38.11%, ginsenoside Rb_1_ accounts for 32.44%, ginsenoside Rd accounts for 4.36%, ginsenoside Re accounts for 5.16%, as shown in Table 1): abnormal XST-1 excluded notoginsenoside R_1_; abnormal XST-2 excluded ginsenoside Rg_1_; abnormal XST-3 excluded ginsenoside Rb_1_; abnormal XST-4 excluded ginsenoside Rd; and abnormal XST-5 halved the content of ginsenoside Rg_1_ and ginsenoside Rb_1_. The components were diluted with fish water according to their content proportion in XST (400 µg/ml).

**TABLE 1:** The content of major compounds in abnormal XST samples.

Batch	Notoginsenoside R_1_ (µmol/L)	Ginsenoside Rg_1_ (µmol/L)	Ginsenoside Rb_1_ (µmol/L)	Ginsenoside RD (µmol/L)	Ginsenoside Re (µmol/L)
Abnormal XST-1	0.00	190.33	116.98	18.40	21.77
Abnormal XST-2	46.51	0.00	116.98	18.40	21.77
Abnormal XST-3	46.51	190.33	0.00	18.40	21.77
Abnormal XST-4	46.51	190.33	116.98	0.00	21.77
Abnormal XST-5	46.51	95.17	58.49	18.40	21.77

Dye working solution consisting of *o*-dianisidine (5.85 mmol/L, dissolved in ethanol), NaOAc (pH = 4.5, adjusted with pH meter (Metter-Toledo International Inc., Zurich, Switzerland)), ultrahigh-purity water, and 30% H_2_O_2_ was mixed at a proportion of 20:5:20:1 before use.

### The Assessment of Anti-Thrombosis Effects Based on Zebrafish Thrombus Model

3dpf zebrafish larvae were used in this experiment, all zebrafish larvae were kept in fish water containing 2.5 µmol/L of PTU in the process of the experiment. The 24-well plates were used for zebrafish larvae placement, double parallel wells were established for each group totally containing 12 fish. For the establishment of the AA-induced zebrafish thrombosis model, staining, fixing, image capture, and quantitative analysis were carried out as reported previously (Qi et al., 2017).

Transgenic LCR-GFP zebrafish were adopted for model validation. After incubation with AA for an hour, the zebrafish were transferred to fish water containing 0.016% tricaine. A video of LCR-GFP zebrafish was taken with a fluorescence microscope (Leica Microsystem Inc., Wetzlar, Germany) with a magnification of ×100, the exposure time was 0.1 s, and the duration was 10 s. The software used for image capture was Andor SOLIS 4.27.30001.0 (Andor Technology Ltd., Belfast, United Kingdom). Each group was photographed under the same conditions.

### Quantitative PCR Detection

Total RNA extraction from 20 zebrafish larvae per group was performed using an Ultrapure RNA kit (Beijing ComWin Biotech Co.,Ltd., Beijing, China) according to the manufacturer’s protocol. Then RNA were reverse transcribed to cDNA with the HiFiScript cDNA Synthesis Kit (Beijing ComWin Biotech Co.,Ltd., Beijing, China). The qPCR was performed by using an UltraSYBR Mixture (Beijing ComWin Biotech Co.,Ltd., Beijing, China) in the Bio-Rad CFX96 real-time system (Bio-Rad Laboratories Inc., California, United States), and the samples were isolated from three independent experiments. *Ef1a* served as the housekeeping gene in each sample. The relative expression of all genes was calculated by 2 ^−ΔΔCt^. All primers sequences are listed in Table 2.

**TABLE 2:** Sequences of primer pairs used in the qPCR.

Gene	Forward primer	Reverse primer
*fga*	5′-CAT​TCA​CTG​CTC​TGC​CTG​TTC-3′	5′-CGC​CTC​TAG​GGT​TCA​CCA​C-3′
*fgb*	5′-CGT​TGG​GAC​GAC​TAC​CGA​AG-3′	5′-AAT​ACG​GTC​ATT​GCC​CAG​CC-3′
*ptgs2a*	5′-TCC​ACA​GAG​GAG​CAG​TCT​CA-3′	5′-AAT​GTG​CCC​CAG​ATC​CAC​TC-3′
*ptgs2b*	5′-TTG​TTG​CTC​CCA​TCC​CTG​TC-3′	5′-GAA​ACT​CGG​GTG​TTG​TGC​AG-3′
*ptgis*	5′-AAC​CTC​CGC​CTG​CTT​ATG​AC-3′	5′-GCG​CCG​AAC​ACT​GTC​AGA​TA-3′
*tbxa2r*	5′-GCG​ACT​ACG​AGG​TGG​AGA​TG-3′	5′-AGA​GCA​GCA​GGT​ATC​GAA​CG-3′
*vwf*	5′-TGG​ATT​GTG​TCA​GTC​TGG​GC-3′	5′-GAC​TCG​TAA​GGC​CTG​CTG​TT-3′
*ef1a*	5′-AGA​AGG​CTG​CCA​AGA​CCA​AG-3′	5′-AGA​GGT​TGG​GAA​GAA​CAC​GC-3′

### HPLC Analysis of XST

Normal XST samples, notoginsenoside R_1_, ginsenoside Rg_1_, ginsenoside Re, ginsenoside Rb_1,_ and ginsenoside Rd were weighed and dissolved in 50% methanol-water solution (*v/v*) in a volumetric flask. The abnormal batches were prepared from five components of XST according to their contents in 10 mg/ml of XST (proportions refer to Reagent Preparation). The content of notoginsenoside R_1_, ginsenoside Rg_1_, ginsenoside Re, ginsenoside Rb_1,_ and ginsenoside Rd in XST (24 and 20) had been quantitatively assessed by the standard curve method.

HPLC was performed by the Agilent 1,100 Series HPLC system (Aglient Co. Ltd., Santa Clara, CA, United States) with an Agilent Zorbax SB-C18 column (250 mm × 4.6 mm, 5 µm) at 28°C. The mobile phase was composed of A (0.01% acetic acid solution) and B (0.01% acetic acid-acetonitrile) with a linear gradient elution program as follow: 0–30 min, 19–21% B; 30–35 min, 21–28% B; 35–41 min, 28–32% B; 41–52 min, 32–32% B; 52–70 min, 32–53% B; 70–80 min, 53–90% B. The injection volume was 20/5 µL. A 10 min equilibration time was adopted between HPLC runs. The flow rate was 1.0 ml/min and the detection wavelength was set at 203 nm.

### Data Analysis

The heart RBCs intensity was assessed by Image-Pro Plus 6.0 (Media Cybernetios Inc., Rockvile, MD, United States). Differences of RBCs between groups were assessed by one-way analysis of variance (ANOVA), difference of inhibition rates between normal and abnormal XST groups was assessed by *t*-test, analysis was performed by GraphPad Prism six software. Peak areas were qualitatively analyzed by the Agilent ChemStation for LC Systems (Rev. B. 04. 03) (Aglient Co. Ltd., Santa Clara, CA, United States).

## Results

### The Anti-Platelet Aggregation Efficacy of XST Based on AA-Induced Zebrafish Thrombosis Model

**FIGURE 1:**
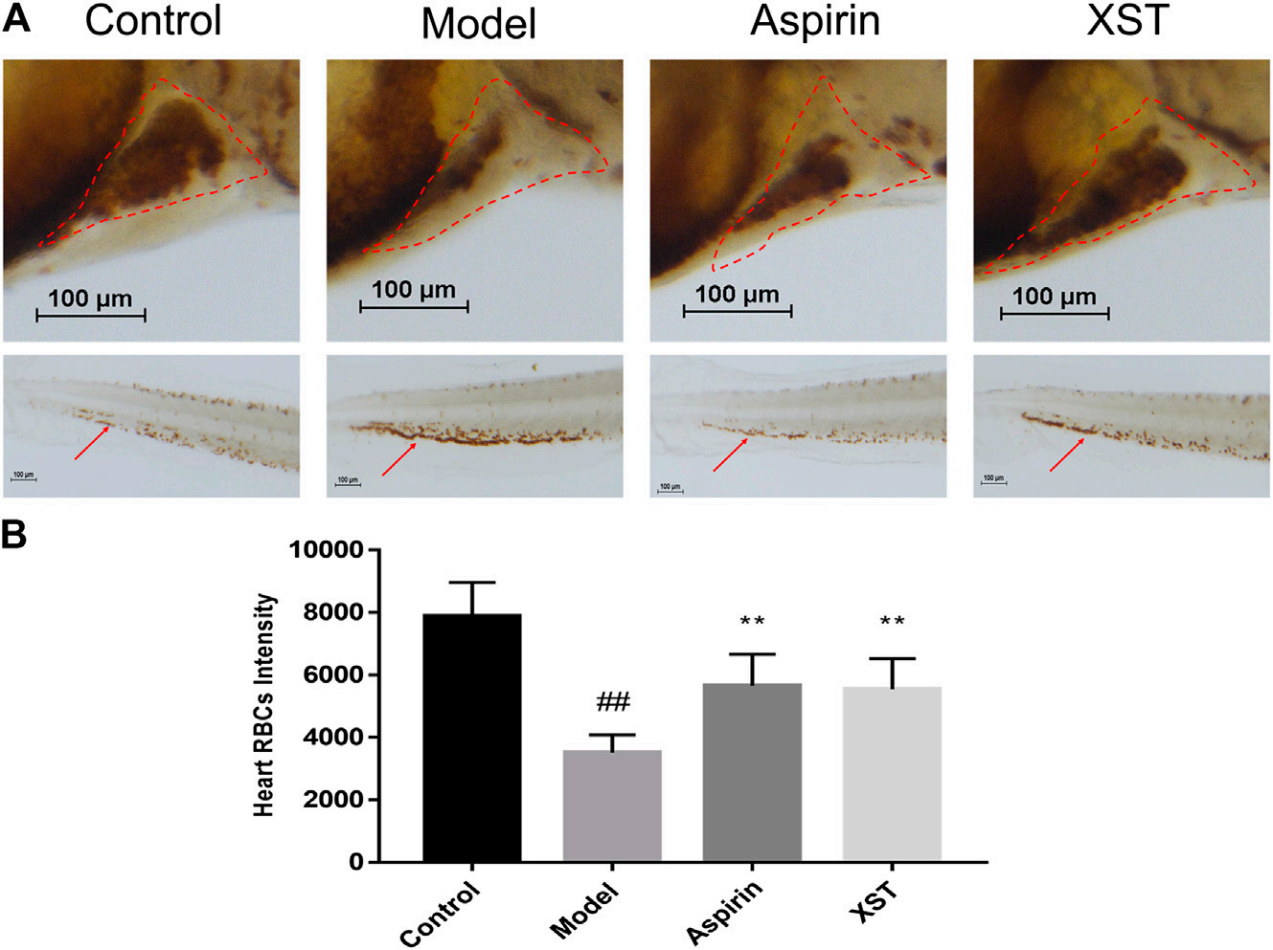
**(A)** Zebrafish were stained with *o*-dianisidine in the control group, model group (AA 40 µmol/L), aspirin group (AA 40 µmol/L + aspirin 22.5 µg/ml), and XST group (AA 40 µmol/L + XST 400 µg/ml), RBCs in heart were marked by red dashed lines, caudal venous thrombus were guided by red arrows; **(B)** quantitative analysis measured the intensity of RBCs of zebrafish in heart area. The error bars represent the standard deviation of means, n = 10. All data were expressed by the mean ± SD, ^##^ *p* < 0.01 vs. control group; **p < 0.01, *p < 0.05 vs. model group.

Zebrafish were stained with *o*-dianisidine, images were acquired through a stereomicroscope and quantified by an image software. As shown in representative images (Figure 1A), compared with the control group, the RBCs were significantly decreased in the heart area of zebrafish in the model group (marked by the red dashed line), apart from this, the accumulation of RBCs in the caudal vein of zebrafish (marked by the red arrow) was significantly enhanced. These results indicated that the AA-induced zebrafish thrombosis model was successfully established. Aspirin is a well-known anti-platelet aggregation drug (Warner et al., 2011). Our results showed that it had outstanding efficacy on zebrafish with thrombus at 22.5 µg/ml. XST also exerted an anti-thrombotic effect at 400 µg/ml, it significantly inhibited RBCs aggregation in caudal venous and recovered the quantity of RBCs in the heart of AA-treated zebrafish. The results of quantitative analysis are exhibited in Figure 1B.

**FIGURE 2:**
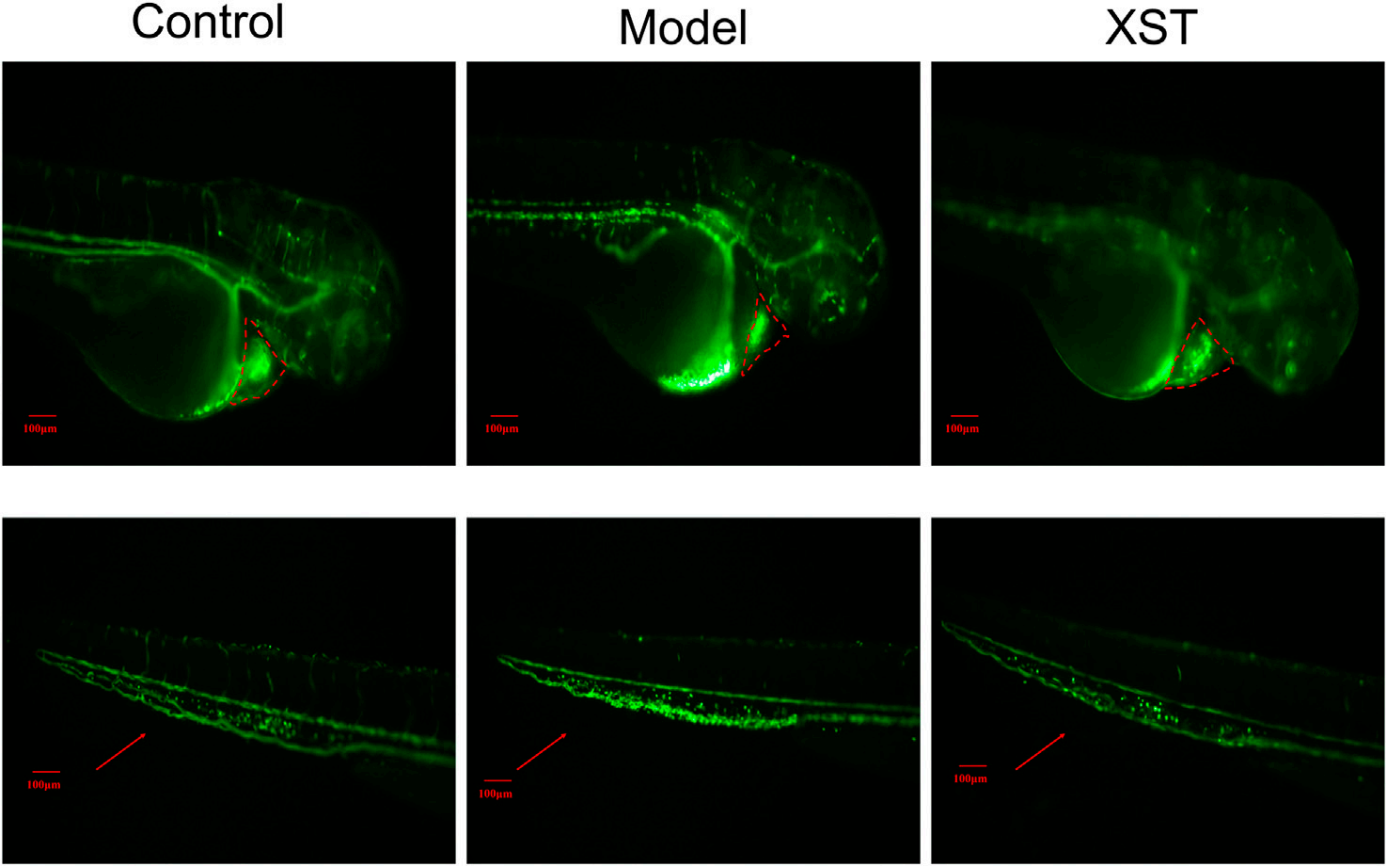
Transgenic LCR-GFP zebrafish in the control group, model group (AA 40 µmol/L), and XST group (AA 40 µmol/L + XST 400 µg/ml), RBCs in heart were marked by red dashed lines, caudal venous thrombus were guided by red arrows. n = 10.

**FIGURE 3:**
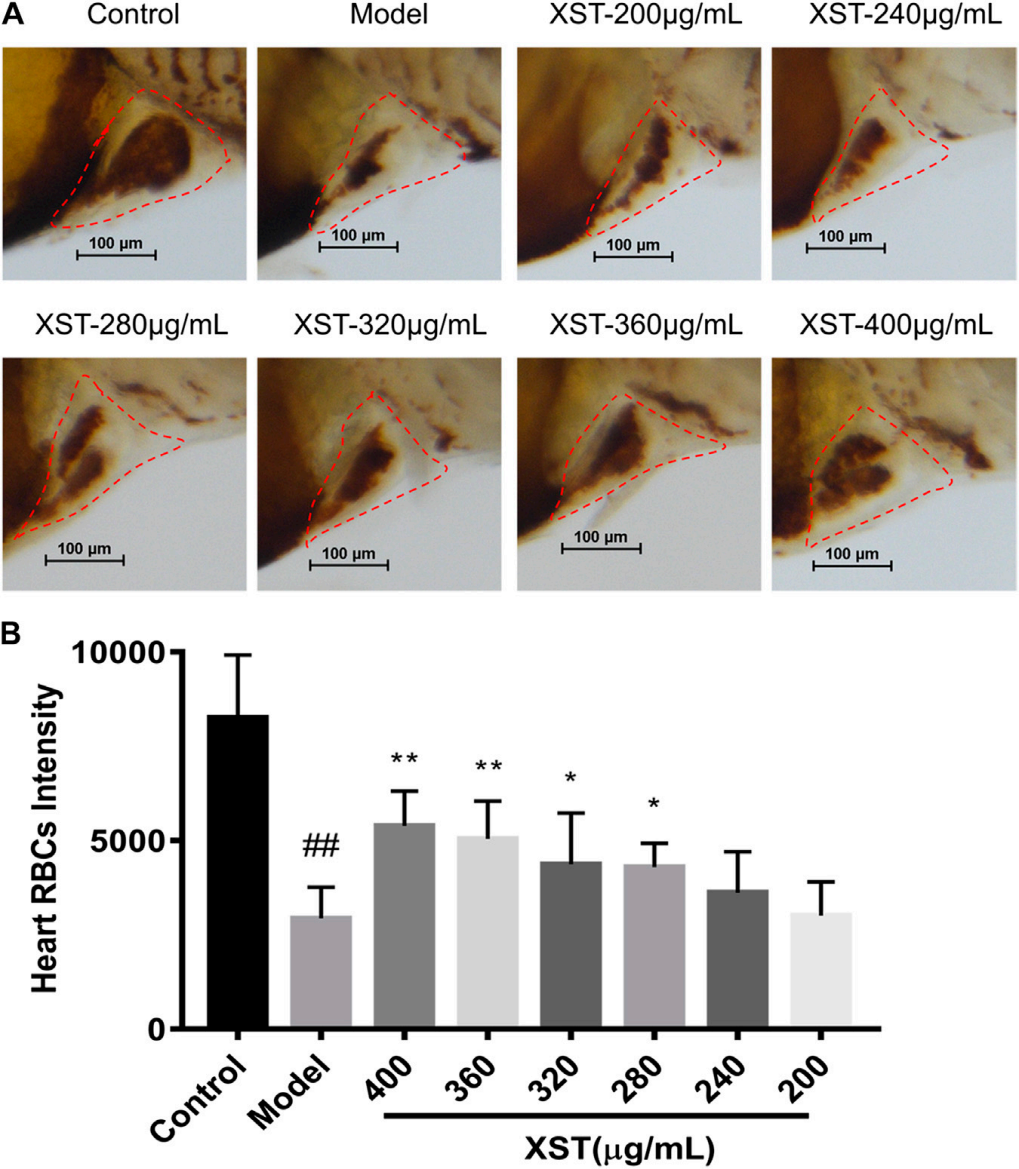
**(A)** Zebrafish were stained with *o*-dianisidine in the control group, model group (AA 40 µmol/L), and XST groups of different concentrations (AA 40 µmol/L + XST 200, 240, 280, 320, 360, and 400 μg/ml), RBCs in heart were marked by red dashed lines; **(B)** quantitative analysis measured the intensity of RBCs of zebrafish in the control group, model group (AA 40 µmol/L), and XST groups of different concentrations (AA 40 µmol/L + XST 200, 240, 280, 320, 360, and 400 μg/ml) in the heart area. The error bars represent the standard deviation of means, n = 10. All data were expressed by the mean ± SD, ^##^ *p* < 0.01 vs. control group; **p < 0.01, *p < 0.05 vs. model group.

In order to verify the results obtained above, LCR-GFP (locals control region-green fluorescence protein) zebrafish with GFP-labeled hemoglobin were adopted for further study. The RBCs of thrombotic zebrafish accumulated in the caudal vein, and the blood flow was discontinuous and slow, furthermore the RBCs in the heart were decreased. The situation was improved in the XST treated group. Representative images were obtained by video recording and are shown in Figure 2. In addition to this, XST could restore RBCs intensity in thrombotic zebrafish dose-dependently, the minimum effective dose was 280 µg/ml (Figure 3B). Representative images are shown in Figure 3A **.**

**FIGURE 4:**
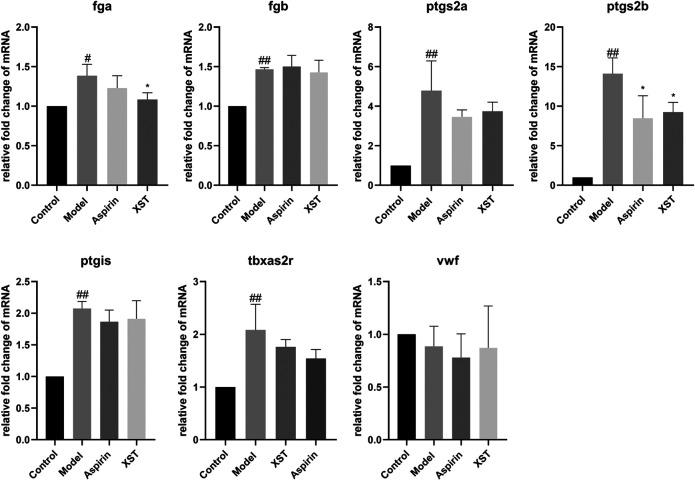
Relative fold change of mRNA of *fga*, *fgb*, *ptgs2a*, *ptgs2b*, *ptgis*, *tbxa2r,* and *vwf* in the control group, model group (AA 40 µmol/L), aspirin group (AA 40 µmol/L + aspirin 22.5 µg/ml), and XST group (AA 40 µmol/L + XST 400 µg/ml), n = 20. All data were expressed by the mean ± SD, ^##^ *p* < 0.01, ^#^ *p* < 0.05 vs. control group; *p < 0.05 vs. model group.

To further explore the potential mechanism of the antithrombotic effect of XST, the relative expressions of seven genes which are related to thrombosis were detected by qPCR. Compared with the control group, the relative expression levels of *fibrinogen alpha chain* (*fga*), *fibrinogen beta chain* (*fgb*), *prostaglandin-endoperoxide synthase 2a* (*ptgs2a*), *prostaglandin-endoperoxide synthase 2b* (*ptgs2b*), *prostaglandin I2 synthase* (*ptgis*) as well as *thromboxane A2 receptor* (*tbxa2r*) in the model group were significantly increased. It was noteworthy that the relative expression levels of *fga* and *ptgs2b* were obviously decreased in the XST group. Besides, the relative expression levels of *ptgs2a, ptgis, and tbxa2r* had a downward trend with the incubation of XST (Figure 4).

### The Anti-thrombotic Effects of Major components in XST

**FIGURE 5:**
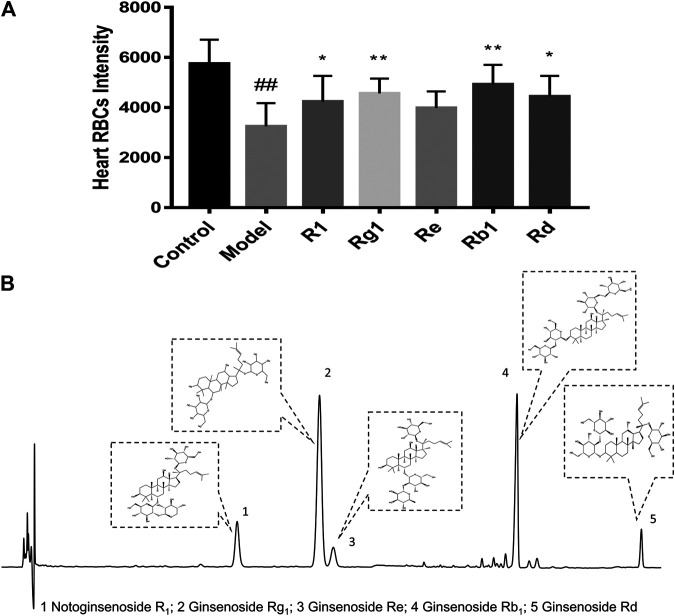
**(A)** Quantitative analysis measured the intensity of RBCs of zebrafish in the control group, model group (AA 40 µmol/L), R_1_ group (AA 40 µmol/L + notoginsenoside R_1_ 200 µmol/L), Rg_1_ group (AA 40 µmol/L + ginsenoside Rg1 200 µmol/L), Re group (AA 40 µmol/L + ginsenoside Re 200 µmol/L), Rb_1_ group (AA 40 µmol/L+ ginsenoside Rb_1_ 200 µmol/L), and Rd group (AA 40 µmol/L + ginsenoside Rd 100 µmol/L). Quantitative analysis was executed to measure the intensity of RBCs of zebrafish in the heart area. The error bars represent the standard deviation of means, n = 10. All data were expressed by the mean ± SD, ^##^ *p* < 0.01 vs. control group; **p < 0.01, *p < 0.05 vs. model group; **(B)** HPLC chromatography of XST and chemical structural formulas of notoginsenoside R_1_, ginsenoside Rg_1_, ginsenoside Re, ginsenoside Rb_1,_ and ginsenoside Rd.

Notoginsenoside R_1_, ginsenoside Rg_1_, ginsenoside Re, ginsenoside Rb_1,_ and ginsenoside Rd were the major active components of XST. To explore the anti-thrombotic components of XST, notoginsenoside R_1_, ginsenoside Rg_1_, ginsenoside Re, ginsenoside Rb_1_ (200 µmol/L), and ginsenoside Rd (100 µmol/L) were administered to thrombotic zebrafish. As shown in Figure 5A, the results indicated that notoginsenoside R_1_, ginsenoside Rg_1_, ginsenoside Rb_1_, and ginsenoside Rd had significant anti-thrombotic effects. The peaks of all five components in the HPLC chromatogram and corresponding chemical structural formulas are given in Figure 5B.

### Comparison of Components Content and Anti-Thrombotic Efficacy of two Batches XST

**FIGURE 6:**
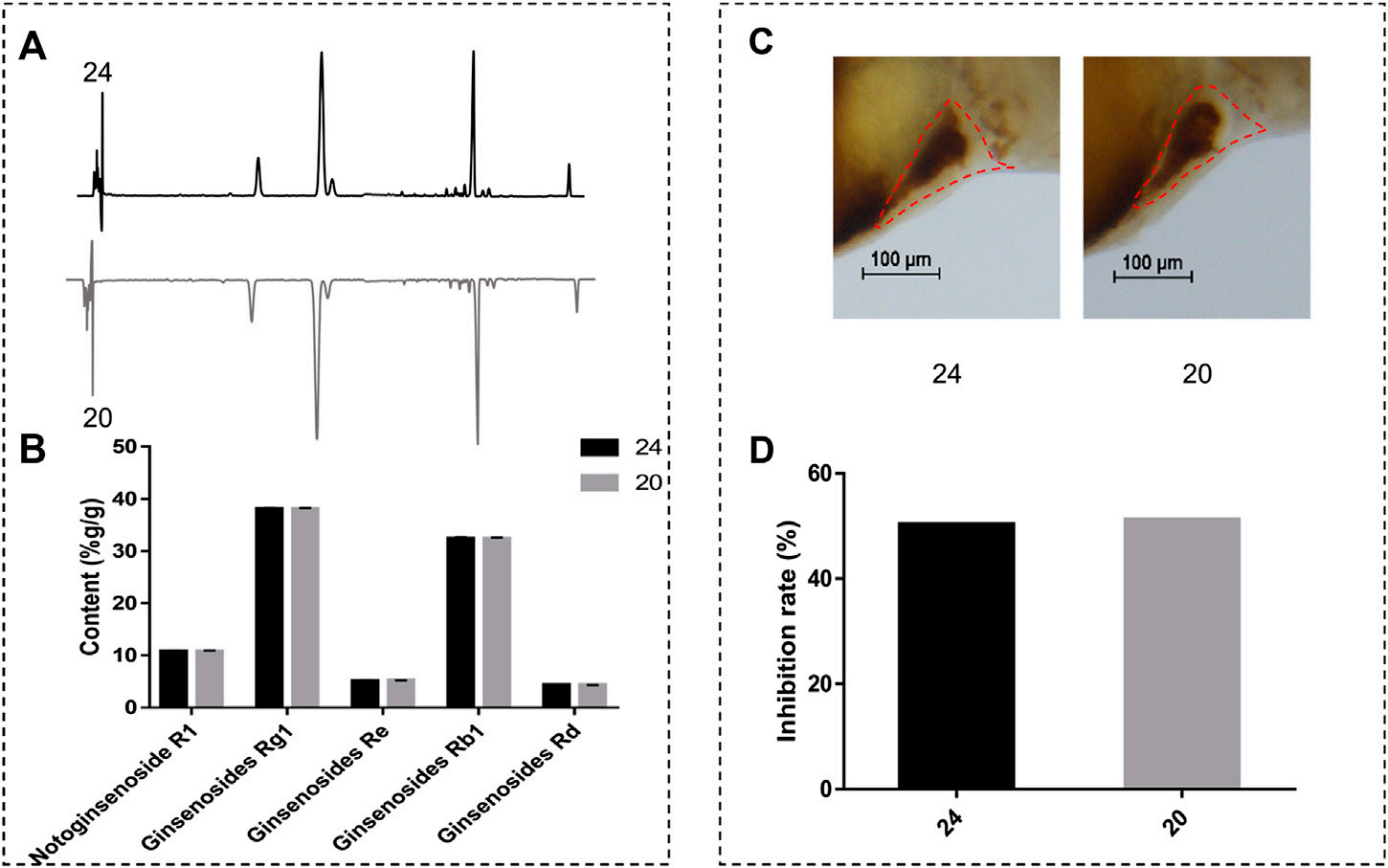
**(A)** The chromatograms of XST; **(B)** content analysis results of notoginsenoside R_1_, ginsenoside Rg_1_, ginsenoside Re, ginsenoside Rb_1,_ and ginsenoside Rd of two batches of XST; **(C)** representative images of two batches of XST treated thrombotic zebrafish, RBCs were marked by red dashed lines; **(D)** inhibition rates of two batches of XST.

In order to investigate whether anti-thrombotic effects were consistent with chemical analysis, we compared the difference of components content and anti-thrombotic efficacy of two batches of normal XST (20 and 24). HPLC was employed to determine the compounds content between two batches of XST, the chromatograms are shown in Figure 6A. The peaks of notoginsenoside R_1_, ginsenoside Rg_1_, ginsenoside Re, ginsenoside Rb_1_, and ginsenoside Rd were qualitatively analyzed. As shown in Figure 6B, the content of major components in different batches were similar. Anti-thrombosis rate was calculated by the formula (Qi et al., 2017) and expressed in inhibition rate. The inhibition rates were counted by measuring RBCs intensity of the heart area in XST treated thrombotic zebrafish quantitatively (Figure 6D), representative images are shown in Figure 6C. The results indicate that the anti-thrombotic effect of two batches of XST were in accordance with the chemical analysis results.

### An Application to Assess Batch-to-Batch Consistency of XST on Zebrafish Thrombosis Model

**FIGURE 7:**
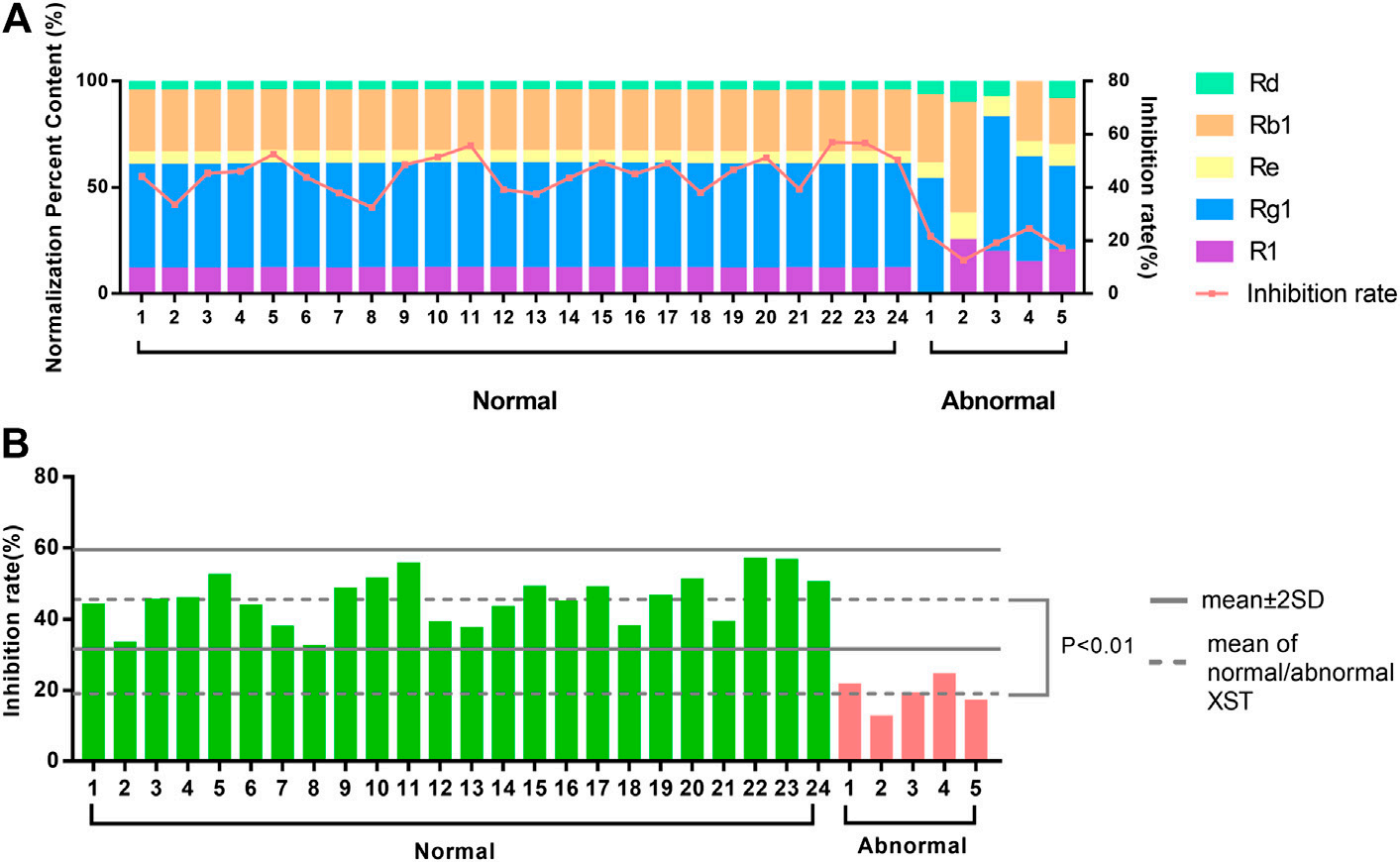
**(A)** The distinction between area normalization percent content and inhibition rates of 24 normal batches of XST and five abnormal batches of XST. **(B)** The distinction between the inhibition rates of 24 normal batches of XST and five abnormal batches of XST.

To explore whether the zebrafish thrombosis model was applicable to assess the quality and batch-to-batch consistency, the 24 normal batches of XST and five abnormal batches of XST were evaluated by both HPLC and the zebrafish thrombosis model. The relative percentages of notoginsenoside R_1_, ginsenoside Rg_1_, ginsenoside Rb_1_, ginsenoside Re, and ginsenoside Rd in each XST sample were expressed as percent content by a peak area normalization method. As shown in Figure 7A, the area normalization percent content in normal batches were relatively consistent, while abnormal batches showed tremendous difference in components percentages. The results of the biological assay were correlated with the HPLC analysis, where abnormal XST had extremely poor anti-thrombotic effects compared with normal XST.

There was a significant difference between the average inhibition rates of normal batches and abnormal batches. The values of mean ± 2SD were considered as the limitation of normal fluctuation, nevertheless the inhibition rates of abnormal batches were below this range (Figure 7B). The zebrafish thrombosis model could be applied to evaluate XST effectiveness and discriminate the quality of normal and abnormal batches. For this reason, the zebrafish thrombosis model was appropriate for the batch-to-batch consistency assessment of XST.

## Discussion

The quality evaluation of CM has received more and more attention recently. Batch-to-batch consistency is an important part of CM quality evaluation. Current methods for consistency evaluation of CM are mainly based on chemical analysis, such as near infrared spectroscopy, HPLC fingerprints, etc. Although the conventional chemical analysis method manifests the difference in chemical markers of different batches intuitively, this cannot comprehensively reflect the bioactivity of CM for the complex composition and multifarious function. The biological assay is based on the pharmacological effects of CM, which is more consistent with clinical efficacy. More and more studies had applied pharmacological activity for the quality assessment of CM. Tsang (Tsang et al., 2019) adopted immunological activities for the quality consistency evaluation of *Dictamnus dasycarpus* Turcz. (Dictamni Cortex), an herbal medicine. Li (Li et al., 2019) established a bioassay method to evaluate the anti-platelet aggregation efficacy of 10 *Reynoutria multiflora* (Thunb.) Moldenke (syn. *Polygonum multiflorum*) samples. Therefore, we proposed a consistency assessment of CM injection based on a model organism. In this experiment, the AA-induced zebrafish thrombosis model established by Qi (Qi et al., 2017) was successfully applied to evaluate the batch-to-batch quality of XST. As a common model organism, zebrafish have the advantages of a short breeding cycle, small volume operation, and convenient administration, so they are suitable for the screening of effective ingredients and toxic components in TCM (Liang, 2009). The zebrafish, whose platelet function has many characteristics in common with humans, is an effective model for thrombosis study. AA is an acknowledged platelet agonist (Weyand and Shavit, 2014). Prostacyclin (PGI2) and thromboxanes (TXs) are two major biologically active substances metabolized by AA through the cyclooxygenase pathway. The breaking of the TXA2/PGI2 balance is the start of platelet aggregation and vasoconstriction (Patrono et al., 1984). Our previous work demonstrated that anti-platelet aggregation was one of the primary mechanisms of XST involved in preventing ischemia-reperfusion injury (Zhao et al., 2017). The clinical dosage of XST is 200–400 mg/D. Based on clinical dosage, we found that XST alleviated cerebral I/R injury in rats with dosages of 40 mg/kg and 80 mg/kg (Wang et al., 2015). Besides, XST could improve myocardial energy metabolism in hypoxia/reoxygenation injured H9c2 cells dose-dependently (200–400 µg/ml) (Zhao et al., 2017). It has been proven that PNS could promote angiogenesis in zebrafish at 100–300 µg/ml (Hong et al., 2009). Using the concentration of 400 µg/ml, XST was able to reverse the aggregation in caudal venous and enhance the RBCs intensity of thrombotic zebrafish significantly (Figure 1). XST improved the blood flow and caudal vein RBCs accumulation of thrombotic transgenic LCR-GFP zebrafish **(** Figure 2). Furthermore, XST could resist AA-induced zebrafish thrombosis in a dose-dependent manner (200–400 µg/ml) (Figure 3). Therefore, we selected 400 µg/ml of XST as a dosage for the quality evaluation experiments.

During the development of thrombosis, the coagulation cascade is an important process, which contains a series of coagulation factors. The activation of coagulation factors will cause the formation of fibrin. The *fga* gene, which encodes fibrinogen, is one of the key genes in the coagulation cascade (Vo et al., 2013). Fibrinogen can interact with the platelet glycoprotein IIb-IIIa to cause platelet aggregation (Farrell et al., 1992). In this study, the expression of *fga* was greatly increased after AA induction, while it was significantly decreased with XST treatment. The results suggested that XST may inhibit the platelet aggregation by reducing the expression of *fga*. *Ptgs2a* and *ptgs2b* are homologous genes of prostaglandin-endoperoxide synthase 2 (*ptgs2*) in mammalian (Ishikawa et al., 2007), which play a key role in inflammatory reaction (Hellmann et al., 2015). Our results showed that AA increased the expression of *ptgs2a* and *ptgs2b* in zebrafish, while XST downregulated the expression of *ptgs2b*, which implied that XST might have an anti-thrombotic effect through ameliorating inflammatory response (Figure 4).

Notoginsenoside R_1_, ginsenoside Rg_1_, ginsenoside Rb_1_, ginsenoside Rd, and ginsenoside Re occupied approximately 85% of the total ingredients in XST. Our results indicated that notoginsenoside R_1_, ginsenoside Rg_1_, ginsenoside Rb_1_, and ginsenoside Rd had significant anti-thrombotic effects in the AA-induced zebrafish thrombosis model (Figure 5). Studies had been reported that PNS, notoginsenoside R_1_, ginsenoside Rg_1,_ and ginsenoside Rb_1_ could inhibit venous thrombosis induced by photochemical reaction (Wang et al., 2008a), ginsenoside Rg_1_ was able to inhibit the activation of platelets and thrombosis of arterial, those results were in line with ours (Zhou et al., 2014).

In order to compare the content of major components and anti-thrombosis rate batch-to-batch, we selected two normal batches of XST for content analysis and anti-thrombotic efficacy detection (Figure 6). Anti-thrombotic efficacy was expressed in inhibition rate. The results showed that the component content and inhibition rates of the two batches of XST were very similar, which indicated that the anti-thrombotic effects of the two batches of XST were in accordance with the chemical analysis results.

To verify whether the zebrafish thrombosis model could distinguish normal XST and abnormal XST accurately, we prepared five abnormal batches of XST according to the ratio of the content of five main components. Each abnormal batch was obtained by reducing or excluding the content of one or two components. The 24 normal batches of XST and five abnormal batches of XST were analyzed by HPLC. The relative content of components in each sample was determined by the normalization method. The components percentages in normal batches were stable, while significant variation was observed in abnormal batches, implying the difference between normal and abnormal samples in chemical composition. The zebrafish thrombosis model was used to detect the inhibition rates of these batches and check whether the results of the two detection methods were consistent. The results showed that the inhibition rates were in accordance with the components percentages. Furthermore, the values of mean ± 2SD could distinguish the abnormal batches from the whole batches, thus the zebrafish model could significantly distinguish normal and abnormal XST. It was noteworthy that the deficiency of ginsenoside Rg_1_ and ginsenoside Rb_1_ causing a relatively great change of inhibition rates.

## Conclusion

In this study, we proved the antithrombotic effects of XST and its potential mechanism. In addition, the inhibition rates of XST in the zebrafish thrombosis model were consistent with the relative percentages of main active compounds in normal or abnormal batches of XST. In short, the study provided a bioassay-based approach to evaluating the batch-to-batch consistency of XST by a zebrafish thrombosis model.

## Data Availability

The raw data supporting the conclusions of this article will be made available by the authors, without undue reservation.
